# Light-activated insulin receptor modulates neuronal plasticity and cerebellar-driven behavior

**DOI:** 10.1016/j.isci.2026.116615

**Published:** 2026-07-02

**Authors:** Bianca Preissing, Franziska Sackel, Marija Trajkovic-Arsic, Lennard Rohr, Anna-Lena Linke, Melanie Bewerunge-Hudler, Sven-Thorsten Liffers, Jens Thomas Siveke, Stefan Herlitze, Ida Siveke

**Affiliations:** 1 Department of Zoology and Neurobiology, Ruhr-University Bochum, 44780 Bochum, Germany; 2 Bridge Institute of Experimental Tumor Therapy, West German Cancer Center, University Hospital Essen, University of Duisburg-Essen, 445147 Essen, Germany; 3 Division of Solid Tumor Translational Oncology, German Cancer Consortium (DKTK, partner site Essen) and German Cancer Research Center, DKFZ, 69120 Heidelberg, Germany; 4 Microarray-Core Facility, German Cancer Research Center, DKFZ, 69120 Heidelberg, Germany

**Keywords:** optogenetics, insulin signaling, neuronal plasticity, behavior, cerebellum

## Abstract

We have developed an optogenetic tool called InLOV—an insulin receptor fused to a LOV domain—that enables activation of the insulin receptor signaling pathway via light-induced phosphorylation of its intracellular domains. Light activation of InLOV promotes insulin-induced neuronal plasticity in the mouse cerebellum and enhances cerebellar-driven self-motion behavior. Thus, InLOV enables optogenetic modulation of insulin receptor phosphorylation, opening new possibilities for disease modeling and therapeutic strategies for pathological insulin signaling in humans.

## Highlights

• Our optogenetic tool, InLOV, enables light-controlled insulin receptor signaling
• Light stimulation of InLOV induces cerebellar neuronal plasticity
• InLOV activation enhances cerebellar-driven self-motion behavior
• New tool for studying and targeting insulin signaling

## Graphical abstract

**Figure:**
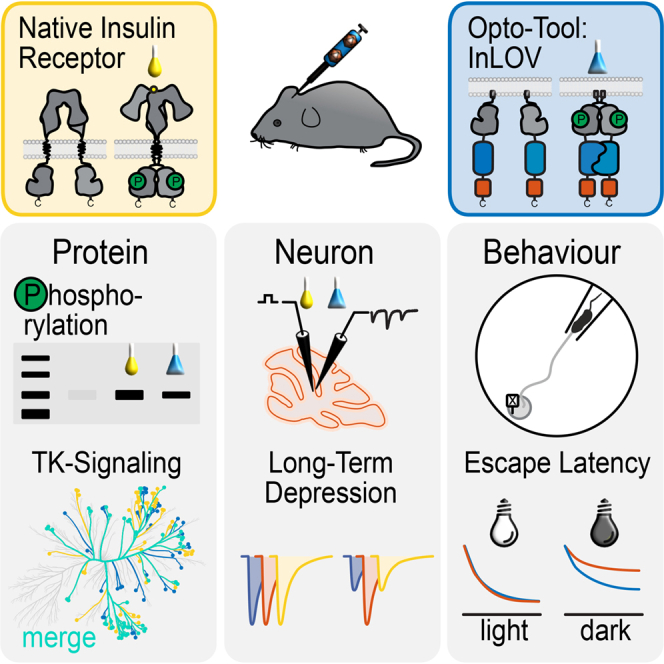

## Teaser

Cell biology; Molecular biology; Neuroscience

## Introduction

Insulin is a key anabolic peptide hormone that regulates numerous metabolic pathways in the body. In the brain, insulin receptor (IR) signaling plays a crucial role in the development and differentiation of neuronal networks by modulating neuronal plasticity.^1^^,^^2^^,^^3^^,^^4^ Insulin resistance is a hallmark of diabetes mellitus and is an important feature in Alzheimer’s disease and related dementias.^4^^,^^5^ This connection is underscored by evidence showing that individuals with type 2 diabetes mellitus have up to a 60% increased risk of developing Alzheimer’s disease.^6^^,^^7^ Notably, the risk for both diseases rises with age, making them two of the most pressing health challenges of our aging society. Optogenetic therapeutic strategies to modulate insulin signaling have focused on the activation of insulin secretion.^8^^,^^9^ Studying the modulatory role of insulin on specific brain regions has been difficult due to the lack of methods for directly and precisely manipulating insulin signaling.

Insulin signaling in the central nervous system extends far beyond metabolic regulation. The brain expresses substantial levels of IRs, particularly in memory-related regions including the hippocampus and prefrontal cortex, as well as in the cerebellum.^1^^,^^2^^,^^3^^,^^10^ Recent studies have demonstrated that insulin modulates synaptic plasticity such as long-term depression (LTD), which is fundamental for learning and memory.^3^^,^^11^ In the cerebellum, IRs are expressed at high levels in Purkinje cells (PCs),^12^ yet the precise role of insulin signaling in cerebellar plasticity and behavior remains incompletely understood.

Several light-activated tyrosine kinase receptors—such as tropomyosin kinase receptors and epidermal or neuronal growth factor receptors—have been engineered and tested in cell culture^13^^,^^14^^,^^15^ and more recently investigated *in vivo*.^16^^,^^17^^,^^18^ However, optogenetic light-activation of IRs has not yet been investigated, despite growing evidence of the involvement of insulin in diverse neuronal processes.

The cerebellum presents a particularly compelling target for investigating insulin signaling, given its well-characterized synaptic plasticity mechanisms and structured neuronal architecture.^19^ LTD at the parallel fiber (PF)-Purkinje cell synapse is a key mechanism underlying cerebellar motor learning, and insulin has been shown to modulate this form of plasticity.^20^^,^^21^^,^^22^ However, the temporal dynamics and cell-specific contributions of insulin signaling to cerebellar LTD have not been dissected due to limitations of pharmacological approaches.

To address this, we developed an optogenetic tool—InLOV—that enables cell-specific, spatial, and temporal control of insulin signaling. Here, we demonstrate that InLOV activation induces IR phosphorylation, promotes LTD at PF-PC synapses, and enhances cerebellar-driven spatial navigation in mice.

## Results

### An optogenetic tool to induce insulin signaling: InLOV

**Figure 1:**
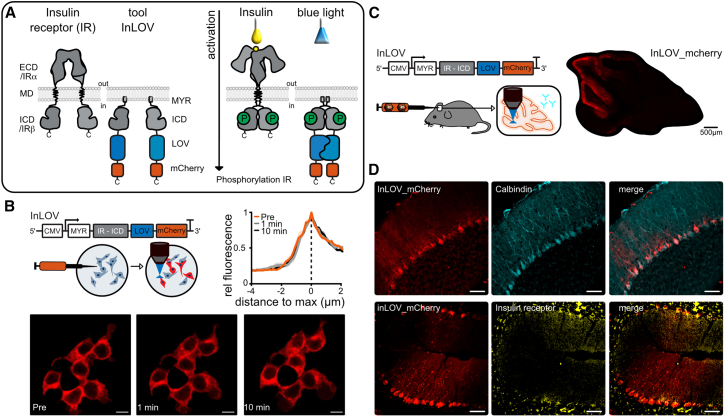
Design and Expression of InLOV (A) Schematic drawing of IR activation and design and function of InLOV. The intracellular tyrosine kinase domain of the IR (ICD/IRb) is fused with a LOV domain. A membrane binding domain, MYR domain, is attached to the N terminus and fluorescent tag (mCherry) fused to the C terminus. (B and C) Scheme of experimental procedure for HEK tsA201 cell imaging and InLOV-expression pre and post (1 min/10 min) blue light stimulation (mean over distance to max, shaded area indicates standard error; *n* = 11). Scale bars, 10 μm; (C) scheme of experimental procedure for cerebellar expression of the constructs and immunostaining. (D) Expression of InLOV and IRs in the cerebellum. Scale bars, 50 μm.

IRs are transmembrane tyrosine kinases consisting of two monomers, each of which has an α and β subunit linked by disulfide bonds. The two extracellular α subunits contain the insulin binding sides, while the β-subunits form the transmembrane tyrosine kinases.^23^^,^^24^ Insulin binding to the extracellular domain promotes dimerization of the intracellular domains (ICDs), which activates intrinsic tyrosine kinase activity and subsequent receptor autophosphorylation (Figure 1A, left). To enable light-dependent control of IR signaling, we developed an optogenetic tool, called InLOV, consisting of the intracellular tyrosine kinase domain of the IR (In) fused to a light-sensing oxygen-voltage (LOV) domain (Figure 1A right). Upon blue light stimulation, the LOV domain promotes homodimerization of the ICDs, mimicking insulin-induced activation and receptor autophosphorylation. InLOV is based on previously developed light-activatable epidermal growth factor receptor tyrosine kinases.^20^^,^^25^ The construct is anchored to the plasma membrane via a myristoylation domain (MYR). A fluorescent protein (mCherry) is fused to the C terminus of InLOV, enabling visualization of expression levels and subcellular localization. HEK tsA201 cells expressing InLOV exhibit a stable fluorescent mCherry signal during blue light stimulation (Figure 1B) not exclusively at the membrane but near it.

To demonstrate the expression of our tool in neuronal tissue, we created viruses expressing either InLOV and, as a control, mCherry alone, and injected them into the cerebellum of mice (Figure 1C). High levels of InLOV expression were detected in PCs within the injected brain regions (Figure 1D), overlapping with the endogenous extracellular α subunit of the IRs.

### Light activation of InLOV induces IR phosphorylation in neuronal tissue

**Figure 2:**
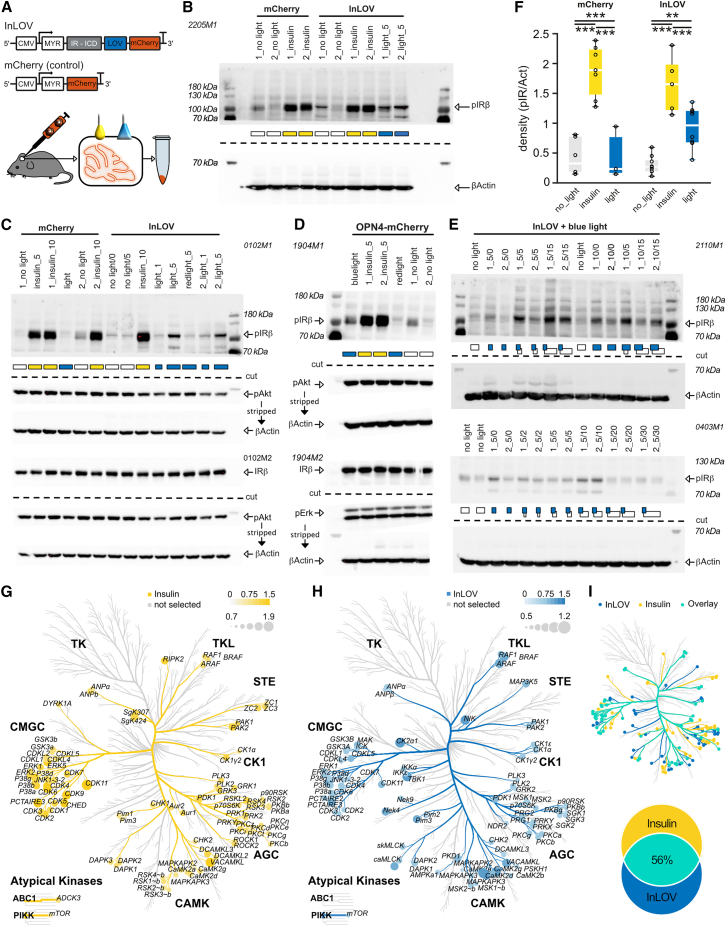
Light activation of InLOV induces IR phosphorylation in cerebellar slices (A) Scheme of the experimental procedure for the molecular analysis. (B) Western blot analysis of the intracellular phosphorylated IRβ-tyrosine kinase domain (pIRβ-) and βActin protein levels after blue light or pharmacological stimulation of InLOV- or mCherry-expressing neurons. (C) Western blot analysis of the pIRβ and βActin protein levels as well as the unphosphorylated IRβ domain and phosphorylated AKT (pAKT) protein after light or pharmacological stimulation of InLOV- or mCherry-expressing neurons in cerebellar slices. (D) Western blot analysis of the pIRβ, pAKT, βActin, IRβ, and pErk1/2 after light and pharmacological stimulation of OPN4_mCherry expressing neurons in cerebellar slices. (E) Protein expression (pIRβ and βActin) of InLOV expressing neurons after blue light stimulation (5 of 10 min) followed by varying durations of darkness. (F) Boxplot of the western blot analysis of pIRβ expression. Dots indicate single data (n_mCherry_nolight/insulin/light = 6/7/3; n_InLOV_nolight/insulin/light = 9/5/8). Band intensities were quantified by densitometry while integrated density was normalized to the loading control (βActin) (repeated ANOVA followed by multi comparison; F_mCherry_[2, 21] = 29, 49; *p* < 0.001; F_InLOV_[2, 25] = 7, 28; *p* = 0,0014, no light_insulin *p* = 0.0024, no light_light *p* = 0.0017). (G–I) The top 87 kinases (median final score for insulin >1, 3) were overlayed on the kinome phylogenetic tree highlighting the kinases that are enriched after insulin application for 30 min (G) or light activation on InLOV expressing cerebellar slices for 10 min (H). (I) Overlay of the two experiments showing 56% commonly enriched kinases (turquoise).

To demonstrate the ability of our tool to induce IR phosphorylation in neuronal tissue using light, we injected viruses expressing either InLOV and, as a control, mCherry alone, and measured IR phosphorylation by western blot analysis (Figure 2A). Blue light stimulation (5 min, 470 nm) of cerebellar slices expressing InLOV increases the phosphorylation of the intracellular IRβ-tyrosine kinase domain, as does the application of insulin to slices expressing either mCherry or InLOV (Figures 2B–2F). Application of insulin induced stronger phosphorylation because, unlike endogenous IR, InLOV is not expressed throughout the entire cerebellar slice, as can be seen in Figure 2B. In contrast, light stimulation of PCs expressing mCherry or OPN4-mCherry, a light sensitive GPCR, does not enhance IRβ phosphorylation (Figure 2D). Consistent with the published phosphorylation kinetics of IRs^26^^,^^27^^,^^28^ the light-induced phosphorylation of the intracellular IRβ-tyrosine kinase domain was maximal at around 5 to 15 min after light application and persisted for at least 15 min after stimulation (Figure 2E). However, even without light or insulin stimulation, we observed phosphorylation of IRβ in all conditions (mCherry, InLOV, and OPN4 expression), indicating basal phosphorylation in the cerebellum. Furthermore, due to already high baseline expression levels, western blot analysis failed to detect differences in the expression levels of the downstream insulin signaling markers pAkt or pErk (Figures 2C and 2D). Therefore, more sensitive kinome profiling of IR downstream signaling was performed comparing the activation by insulin or InLOV to control conditions (Figures 2G–2I). This analysis revealed multiple serine/threonine phosphorylation within the ERK signaling cascade (see CMGC kinase family tree, Figures 2F and 2G). The top kinases were overlayed on the kinome phylogenetic tree, highlighting those enriched following insulin application to activate endogenous IRs or light-activation of InLOV expressing PCs and showing that 56% of the kinases are commonly enriched (Figure 2I). Although InLOV does not fully recapitulate the entire signaling network induced by endogenous IR signaling, it initiates large components of insulin signaling.

### InLOV activation modulates LTD in cerebellar slices

**Figure 3:**
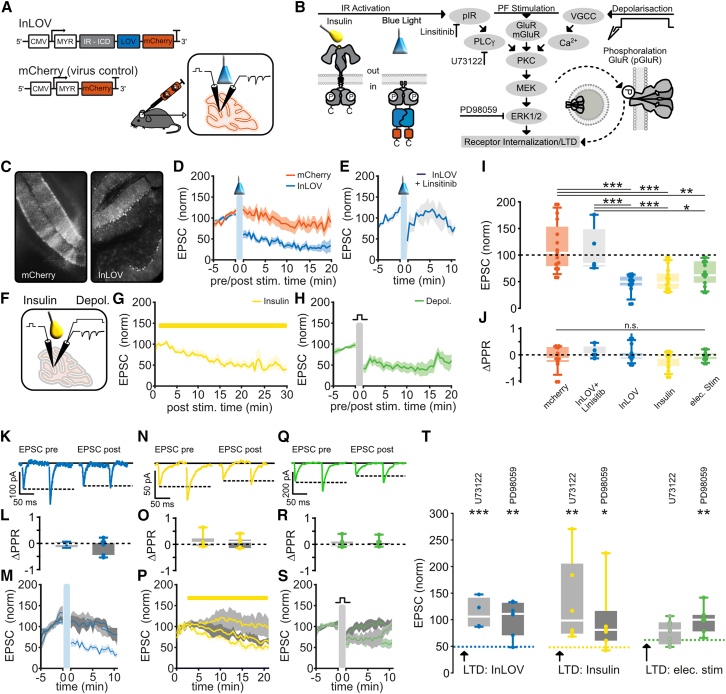
InLOV activation modulates LTD in cerebellar slices (A) Simplified graphic representation of the intracellular mechanism that triggers LTD. (B) Scheme of the experimental procedure for light-induced LTD by cerebellar slices expressing two different tools (mcherry and InLOV). (C) Expression of the two constructs in PCs of 250 μm-cerebellar slices. (D) EPSCs were evoked by electrical stimulation combined with 470 nm blue light stimulation. EPSCs were normalized to the average pre-EPSCs (–5–0 min). EPSCs before and after blue light stimulation are given as mean over time (shaded area indicates standard error). LTD of post EPSCs are induced by optical stimulation of InLOV (InLOV, *n* = 12, *p* < 0.001) but not light activation of mCherry expressing PCs (*n* = 13, *p* = 0, 74). (E) Linsitinib, a blocker of IR phosphorylation, blocked light-induced LTD of InLOV expressing PCs (*n* = 5, *p* = 0, 69). (F) Scheme of the experimental procedure for insulin or depolarization induced LTD in cerebellar slices. (G and H) LTD of post EPSCs are induced by insulin application (*n* = 14, *p* < 0.001) or depolarization of the PCs (*n* = 11, *p* < 0.001). (I) Multi comparison of the changed in EPSC of all five experiments, shown as a boxplot with single cells overlaid as dots. (Repeated ANOVA followed by multi comparison; F [4, 18] = 6, 35; *p* < 0.001, mcherry-Depol *p* = 0.003, Linitinib-Depol *p* = 0.018, all other combination *p* < 0.001). (J) No changes were found in the PPR. (K–P) InLOV and insulin induced LTD is blocked by U73122(PLC) and PD98059 (pErk) (InLOV/U73122 *n* = 4,/PD98059 *n* = 7; Insulin/U73122 *n* = 5,/PD98059 *n* = 7). (K–P) PD98059 but not U73122 blocked the LTD induced by depolarization (Depol/U73122 *n* = 7; *p* = 0.018/PD98059; *n* = 7). (K, N, and Q) Example EPSCs induced by the paired pulse stimulations shown for the three conditions, stimulus artifacts were removed. No changes were found in the PPR. (T) Multi comparison of the changed in EPSC separated by the type of LTD induction, shown as a boxplot with single cells overlaid as dots (repeated ANOVA followed by multi comparison; F_InLOV_[2, 18] = 6,35; *p* < 0.001, InLOV-U73122 *p* < 0.001, InLOV-PD98059 *p* = 0.004; F_Insulin_[2, 18] = 6,35; *p* = 0.011, insulin-U73122 *p* = 0.003, insulin-PD98059 *p* = 0.028; F_Depol_[2, 18] = 6, 35; *p* = 0.017, Depol-U73122 *p* = 0.28, Depol-PD98059 *p* = 0.006). The median measured LTD (shown in I) is plotted as a dashed line.

*In vitro* studies in the cerebellum have shown that electrical stimulation of the PF inputs, combined with either application of insulin or depolarization of PCs, induces LTD of excitatory postsynaptic currents (EPSCs).^20^^,^^21^ In simple terms, PC depolarization, which is intended to mimic the climbing fiber (CF) input, primarily induces large Ca^2+^ transients through voltage-gated calcium channels, while IR phosphorylation triggers PLCγ activation. Among others, this results in the activation of the protein kinase C (PKC) and ERK (ERK1/2) signaling, and finally, AMPAR internalization (Figure 3A).^20^^,^^29^^,^^30^^,^^31^^,^^32^ Various studies using pharmacological blockers revealed the critical roles of Ca^2+^, PKC and ERK1/2 in the induction of LTD at the PF-PC synapse triggered by CF stimulation, glutamate or insulin.^20^^,^^22^^,^^33^^,^^34^^,^^35^ Based on these findings, we first tested whether light activation of InLOV could induce LTD in the cerebellar cortex. We expressed InLOV (blue), and as a control, mCherry (red) in cerebellar PCs and measured EPSCs using patch-clamp recordings (Figures 3B and 3C). We found that, in PCs expressing InLOV, light activation (470 nm) induces LTD of EPSCs at PF-PC synapses, whereas this does not occur in mCherry-expressing PCs (Figure 3D). Light-induced LTD induction was blocked by Linsitinib, a selective blocker of IR autophosphorylation (Figure 3E). Similarly, both the application of insulin (yellow, Figures 3F and 3G) and the depolarization of PCs (green, Figure 3H) induces significant LTD of EPSCs at PF-PC synapses (Figure 3I). Investigation of the paired pulse ratio (PPR), which remained unaltered and would indicate presynaptic changes, indicated that the observed LTD takes place presynaptically (Figure 3J).

Next, to investigate the underlying signaling pathways of the observed LTD (Figure 3B), which might be activated by IR phosphorylation (InLOV/insulin) or electrical stimulation, we blocked PLCγ (light gray) or ERK1/2 (dark gray) phosphorylation using U73122 or PD98059, respectively. We demonstrate that the LTD induced by InLOV or insulin can be blocked by PLCγ and ERK1/2 inhibitors (Figures 3K–3P). However, LTD induced by electrical depolarization is blocked by the not ERK1/2 inhibitors but not affected by PLCγ inhibition (Figures 3Q–3T). This suggests that LTD induction by InLOV and insulin involves PLCγ and ERK1/2 signaling. However, LTD induction by electrical depolarization is independent of PLCγ activation and involves different intracellular signaling cascades. The PPR remained unaltered (Figures 3L, 3O, and 3R). Together, these findings demonstrate that light activation of InLOV induces LTD at the postsynaptic side of the PF-PC synapse by activating the insulin signaling pathway.

### InLOV activation modulates cerebellar-driven behavior

**Figure 4:**
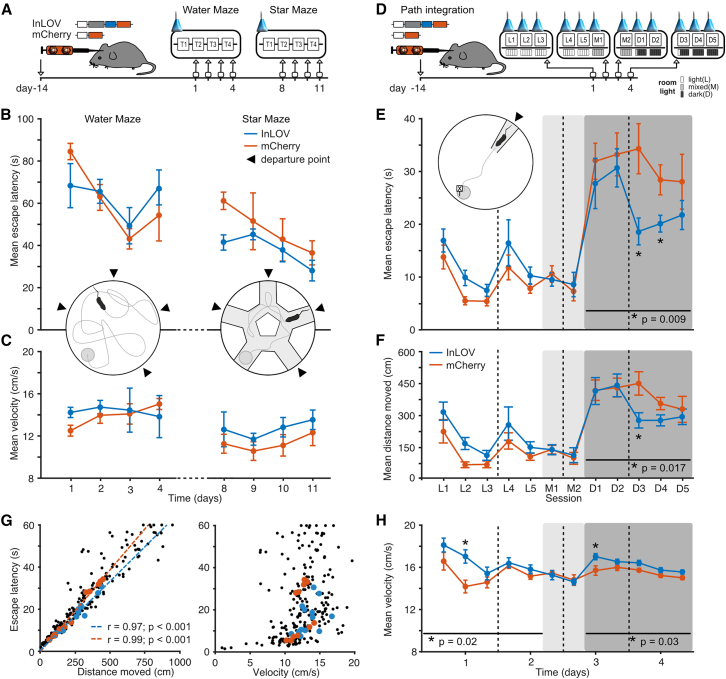
InLOV activation modulates cerebellar-driven behavior (A–C) Performance of InLOV or mCherry expressing mice in the Water Maze and Star Maze test. (A) Protocol for Water Maze (InLOV, *n* = 5 animals, mCherry, *n* = 5 animals) and Star Maze (InLOV, *n* = 4 animals, mCherry, *n* = 5 animals) experiments. (B–C) Escape latency to reach the platform (B) and swimming velocity (C). h-l, performance of InLOV (*n* = 8 animals) or mCherry (*n* = 7 animals) expressing mice in the path integration test. (D) Path integration protocol consisted of light (L1–5), mixed (M1–M2), and dark training sessions (D1–D5) each consisting of five trails. (E–G) Averaged performance of each session shown as mean escape latency (E) or mean distance moved (F); repeated ANOVA followed by multi comparison; escape latency, F(1, 13) = 9, 6; *p* = 0.009; D3, *p* = 0.015, D4, *p* = 0.029; distance moved, F(1, 13) = 7, 4; *p* = 0.017; D3, *p* = 0.026. Error bars indicate SEM, dashed lines different days, gray areas light intensities. (G) Escape latency correlates with distance moved (left) but not with velocity (right). Black dots indicate the single data measured at day 4, blue (InLOV) and red (mCherry) dots the mean data of the different sessions. (H) Swimming velocity; repeated ANOVA followed by multi comparison; light; F(1, 13) = 6, 5; *p* = 0.024; L2, *p* = 0.015; dark; F(1, 13) = 5, 7; *p* = 0.032; D1, *p* = 0.034.

The cerebellum is involved in several aspects of motor learning as well as more cognitive tasks, including spatial memory. Studies using a transgenic mouse strain (L7PKCI), which lacks PKC-dependent LTD at the PF-PC synapse, have suggested the hypothesis that long-term plasticity in the cerebellum plays a critical role in particular forms of motor learning.^34^^,^^36^ Robust behavioral effects are observed when mice must rely on self-motion cues without external information (i.e., when navigating in the dark).^36^^,^^37^ We therefore investigated whether light activation of insulin signaling, triggering LTD at the PF-PC synapse, prior to a behavioral task, improves spatial navigation in mice. InLOV or mCherry was injected into specific areas in the cerebellar cortex (in the hemispheres and the posterior vermis), which have been shown to be critical for motor learning and for retaining information in working memory.^38^^,^^39^ Following recovery, mice were trained to find an escape platform at a constant location in the Water Maze and then in the Star Maze (Figures 4A and 4D). Before each trial, light was applied to the cerebellar cortex via implanted optical fibers. Both groups of mice learned to find the platform, but no significant differences were observed between the two groups in terms of escape latency (Figure 4B) or the swimming velocity (Figure 4C). Next, we performed the path integration task, in which the mice rely on self-motion cues.^40^ In this task, mice were trained to find an escape platform at a constant location with a constant departure point in both light and dark conditions (Figure 4D). Light stimulation was applied to the cerebellum before each training block. In the light, both groups learned to reach the platform equally fast (Figures 4E and 4F). In contrast, InLOV-expressing mice showed improved navigation performance in the dark (Figures 4E and 4F), and the faster escape latencies correlated with smaller distances moved rather than swimming velocity (Figures 4G and 4H).

## Discussion

### In summary, we establish a next generation optogenetic tool called InLOV for precise investigation and modulation of insulin signaling

InLOV extends the growing family of optogenetic receptor tyrosine kinases^14^^,^^15^ to the IR, filling an important gap given the widespread expression of IR in the nervous system and its implicated roles in neuronal plasticity, survival, and metabolism.^1^^,^^2^^,^^3^^,^^4^^,^^5^ Unlike pharmacological approaches, InLOV provides cell-type-specific and temporally precise control of IR signaling, enabling dissection of acute signaling events that are inaccessible through conventional methods. Our kinome profiling demonstrates that InLOV activates a substantial fraction (56%) of the kinases also activated by insulin, confirming that the tool engages physiologically relevant downstream signaling cascades. However, InLOV does not fully recapitulate the complete signaling network induced by endogenous IR activation, which likely reflects the absence of the extracellular ligand-binding domain and the potentially different subcellular localization of the overexpressed construct. We would like to point out, that this is the first study investigating and comparing the kinome of native versus tool-induced intracellular signaling.

Our electrophysiological findings reveal that insulin signaling plays a direct role in the induction of LTD at the PF-PC synapse. The dependence of InLOV-induced LTD on PLCγ and ERK1/2 signaling, but not on Ca^2+^ influx through VGCCs, distinguishes this form of plasticity from classical CF-dependent LTD and suggests that insulin activates a parallel intracellular signaling cascade converging on AMPAR internalization.^20^^,^^21^^,^^22^^,^^33^^,^^34^^,^^41^ These results are consistent with previous pharmacological studies demonstrating insulin-induced LTD in cerebellar slices^20^^,^^21^ and extend them by providing the first evidence for optogenetically controlled insulin-dependent plasticity in intact neuronal circuits.

The behavioral experiments demonstrate that enhancing insulin signaling in the cerebellar cortex improves spatial navigation specifically when mice rely on self-motion cues (path integration in the dark), while general spatial learning in the Water Maze and Star Maze remains unaffected. This dissociation is consistent with previous studies showing that cerebellar LTD deficits preferentially impair path integration rather than allocentric spatial strategies.^36^^,^^37^^,^^42^ The improved performance of InLOV-expressing mice in the dark suggests that insulin-mediated enhancement of PF-PC plasticity facilitates the processing of self-motion information by cerebellar circuits. However, targeting different cerebellar regions may alter the results induced by insulin signaling. A valuable future direction would be to examine how modulating insulin signaling in other cerebellar regions affects distinct behavioral functions. Furthermore, future studies expressing InLOV in hippocampal neurons or hypothalamic circuits could reveal region-specific roles of insulin signaling in memory consolidation and metabolic homeostasis, respectively.

The link between insulin resistance and neurodegenerative disease, particularly Alzheimer’s disease, is increasingly recognized.^5^^,^^6^^,^^7^^,^^43^ Brain insulin resistance has been proposed as a contributing factor to cognitive decline, and intranasal insulin administration has shown promise in clinical trials for Alzheimer’s disease.^44^^,^^45^ Our demonstration that IR signaling directly modulates cerebellar plasticity provides new mechanistic insights into how insulin resistance might contribute to motor and cognitive impairments. InLOV offers a powerful tool for testing whether restoration of insulin signaling in specific brain regions can reverse plasticity deficits in disease models.

Thus, our results open the opportunity for optogenetic activation of insulin pathways not only in the brain, but also in the pancreas, gut, and other tissues, enabling the development of new therapeutic strategies related to impaired insulin signaling.

### Limitations of the study

Several limitations of this study should be acknowledged. First, InLOV consists of the intracellular tyrosine kinase domain of the IR fused to a LOV domain and therefore lacks the extracellular insulin-binding domain. This design means that InLOV activation may not fully recapitulate all aspects of endogenous IR signaling, particularly with respect to receptor conformation and interaction with extracellular binding partners. The subcellular localization of overexpressed InLOV, anchored via a MYR domain, may also differ from that of endogenous IRs, potentially affecting access to specific downstream effectors. Additionally, AAV expression using the CMV promotor in neurons or cells that do not endogenously express IRs might contribute to the observed effects, warranting cell-type-specific investigations in future studies.

Second, this study focused exclusively on cerebellar PCs in the proximal vermis and lateral hemisphere. Whether InLOV-mediated effects on plasticity and behavior generalize to other cerebellum or brain regions where insulin signaling plays important roles, such as the hippocampus or hypothalamus, remains to be determined.

Third, the sample sizes used in this study, while typical for electrophysiological and behavioral investigations, are relatively modest. We did not conduct separate analyses by biological sex, which may be an important variable given known sex differences in insulin sensitivity.^46^ Larger, sex-stratified cohorts would strengthen the generalizability of these findings.

Fourth, we examined only acute and short-term effects of InLOV activation. Long-term consequences of repeated optogenetic IR stimulation, including potential compensatory mechanisms or cellular toxicity, were not investigated and would be important to characterize before considering therapeutic applications.

Finally, the blue light wavelength (470 nm) required for LOV domain activation has limited tissue penetration, necessitating invasive fiber optic delivery. Development of red-shifted optogenetic IR variants would be an important step toward broader applicability and potential clinical translation.^18^^,^^47^

## Resource availability

### Lead contact

Further information and requests for resources and reagents should be directed to and will be fulfilled by the lead contact, Ida Siveke (ida.siveke@rub.de).

### Materials availability

The commercially available material is denoted in the manuscript. Further information and requests for resources and reagents should be directed to and will be fulfilled by the lead contact.

### Data and code availability

The datasets will be shared by the lead contact upon request. All codes that were used for analysis are available upon reasonable request from the lead contacts. Any additional information required to reanalyze the data reported in this study is available from the lead contact upon request.

## Acknowledgments

We thank Michelle Grömke, Brix Mücher, Helin Kenger, and Harald Janovjak for their technical help and critical comments. Project number 316803389-SFB1280/A07, Priority Program SPP1926 (DFG2471/18-2), DFG2471/21-1, DFG2471/23-1 (to S.H.), 492434978-GRK2862/1 (I.S. and S.H.). J.T.S. is supported by the 10.13039/501100012353German Cancer Consortium (10.13039/501100012353DKTK).

## Author contributions

Conceptualization, B.P., S.H., and I.S.; methodology and investigation, B.P., F.S., L.R., M.T.-A., A.-L.L., M.B.-H., S.-T.L., and I.S.; data analysis, B.P., F.S., L.R., M.T.-A., M.B.-H., and I.S.; funding acquisition, J.T.S., S.H., and I.S.; supervision, J.T.S., S.H., and I.S.; writing, S.H. and I.S.

## Declaration of interests

The authors declare no competing interests.

## Declaration of generative AI and AI-assisted technologies in the writing process

During the preparation of the revised manuscript, the authors used Perplexity AI to convert the manuscript from a report to an article. After using this tool, the authors reviewed and edited the content as needed and take full responsibility for the content of the publication.

## STAR★Methods

### Key resources table

**Table:** 

REAGENT or RESOURCE	SOURCE	IDENTIFIER
**Antibodies**		

Insulin receptor beta domain (IRb)	Cell Signaling	#3025S; RRID: AB_2280448
pIRb	Cell Signaling	#3024S; RRID: AB_331253
pAkt	Cell Signaling	#9271; RRID: AB_329825
pErk1/2	Cell Signaling	#4376S; RRID: AB_331772
Insulin receptor alpha domain	abcam	#283689

**Chemicals, peptides, and recombinant proteins**		

QX-314	Tocris Bioscience	# 5369-03-9; RRID: SCR_003689
Linsitinib	MedChemExpress	#HY-10191; RRID: SCR_015306
U73122	MedChemExpress	# HY-13419; RRID: SCR_015306
PD98059	MedChemExpress	# HY-12028; RRID: SCR_01536
Insulin	Sigma-Aldrich	#I9278; RRID: SCR_008988

**Experimental models: Cell lines**		

HEK293-T cells	Dr Deniz Dalkara	N/A

**Experimental models: Organisms/strains**		

Mouse: C57Bl/6	JAX	RRID: IMSRJAX:000664

**Recombinant DNA**		

VfAU1-LOV	Addgene	RRID: Addgene58745
pAAV-CW3SL-eGFP	This paper	N/A
pAAV-CW3SL-mCherry	This paper	N/A
pAAV-CMV-InLOV-mCherry	This paper	N/A

### Experimental model and study participant details

All experiments were conducted with approval of a local ethics committee (Bezirksamt Arnsberg) and the animal care committee of Nordrhein-Westfalen (LANUK, Landesamt für Natur, Umwelt und Klima, Nordrhein-Westfalen, Germany; AZ. 84–02.04.2014.A203 und AZ.81–02.04.2019.A228). Experiments were performed in 2- to 6-month-old male and female C57Bl/6/J mice (JAX stock no. 000664). No sex differences were observed. For *in vitro* and behavioral experiments, animals received viral injections with InLOV-mCherry or mCherry (as control). Mice were kept on a 12/12 days/night cycle with water and food *ad libitum*. For cell culture experiments and AAV virus production human embryonic kidney (HEK) tsA201 cells (Sigma Aldrich, Taufkirchen, Germany) were used.

### Method details

#### Generation of plasmid constructs

The backbone of the generated plasmids emerges from a pAAV-CW3SL-eGFP cloning vector (GenBank accession number: KJ411916.2) to ensure sufficient packaging capacity for adeno-associated virus production.^48^ For the production of the pAAV-CMV-InLOV-mCherry construct (InLOV), the intracelllular kinase domain of a murine insulin receptor (INSR-MOUSE, residue 968–1372 of Uniprot entry P15208) was used after synthetization with mammalian codon optimization and flanked by genes coding for MYR and the LOV domain of *Vaucheria frigida* aureochrome1 (VfAU1-LOV, aureochrome1, residue 204–348 of Vaucheria frigida, A8QW55) (Addgene plasmid #58745; http://n2t.net/addgene:58745; RRID: Addgene58745). mCherry was tagged C-terminally to the InLOV construct as fluorescence marker. The individual DNA sequences were PCR amplified and introduced into the backbone via 16 bp overhangs using InFusion cloning kit (TAKARA). The mCherry control plasmid was generated equally except for removal of the insulin receptor and LOV domain sequences. All constructs generated were verified by sequencing.

#### Cell culture and AAV virus production

Human embryonic kidney (HEK) tsA201 cells (Sigma Aldrich, Taufkirchen, Germany) were maintained in Dulbecco’s modified Eagle’s medium (DMEM), 4.5 g/L D-glucose and supplemented with 10% fetal bovine serum (Gibco) in a cell culture incubator at constantly 37°C under a 5% CO_2_ atmosphere. To produce a recombinant adeno-associated virus serotype 8 (AAV8), the AAV helfer-free system (Agilent Technologies, Santa Clara, CA) was used. HEK tsA201 cells at 80–90% confluence were co-transfected with three different plasmids via polyethyleneimine (PEI): the plasmid encoding the gene of interest, the adenovirus helper plasmid and the plasmid encoding the AAV *rep* and *cap* genes. To harvest viral particles after an incubation period of ∼72 h, transfected cells were separated by centrifugation (1500 rpm, 10 min at 4°C) and resuspended in a lysis buffer (150 mM NaCl, 50 mM Tris-Cl, pH 8.5). While the collected supernatant of the harvested cells was incubated with PEG-8000 (10% final w/v) for 2 h at 4°C on a shaker, cell lysis was supported over 5–7 freeze-thaw cycles. In the next step, centrifugation was performed for 20 min at 3700 x g and 4°C. The clarified supernatant of the lysed cells was used to resuspend the PEG-precipitated pellet. To purify the virus, the obtained resuspension was again incubated with PEG-8000, centrifuged and the pellet resuspended in 50 mM HBS. Chloroform was added at room temperature in a 1:1 ratio, the mixture vortexed for at least 2 min and centrifuged at RT and 370 x g for 5 min. The clarified supernatant was sterile filtered (0.22 μm), concentrated using PEG-8000 for 2 h at 4°C and again centrifuged at 3700 x g for 20 min at 4°C. The concentrated AAV pellet was resuspended in 0.001% PBS-Pluronic F-68, aliquoted and stored at −80°C.

#### Stereotaxic surgery

For virus injection and fiber implantation, mice were deeply anesthetized with isoflurane (initial dose: 5% in 1.1 L/min^−1^ air flow), fixed in a stereotaxic frame (Narishige, Tokyo, Japan) and analgesized by subcutaneous injection of carprofen (2 mg kg^−1^) and buprenorphine (0.1 mg kg^−1^). Anesthesia and body temperature were continuously controlled by an isoflurane vaporizer (1.3–2.0% isoflurane maintenance) and a heating pad. The eyes were protected from drying with a hydrating ointment. Once the animal was verified to be insensitive to pain by toe pinch, the cranial hair was first removed, the scalp cleaned using 70% ethanol and opened via a straight sagittal incision. To provide additional local anesthesia, lidocaine was applied to the skull. For surgeries, craniotomies were made at intended coordinates (1^st^ and 2^nd^, cerebellar hemispheres (Curs I): AP = −6.3; ML = ±1–1.2; DV = 1–3; 3^rd^ posterior vermis (around lobe V): AP = 6; ML = 0; DV = 1–2). For virus injection, AAV was drawn up into a customized glass pipette (GC 15010, Harvard Apparatus) via a syringe and applied using pressure injection. For fiber implantation, the skull bone was additionally treated with OptiBond universal (Kerr, CA, USA) solidified via UV exposure (GDT super 1200 UV light source) and two custom-made optical fibers (200 μm fiber diameter, 0.39 NA; FT200MT, 1.25 mm outer ferrule diameter; Thorlabs, Dortmund, Germany) were implanted in two of the craniotomies respectively (cerebellum: left and right, lowered to 0.8 mm) and attached to the skull using dental Charisma (Heraeus Kulzer, Hanau, Germany). The opened scalp was sewn up and the remaining wound edges attached to the charisma using Histoacryl (B. Braun). After the surgery, mice were placed in individual cages and treated with analgesia for 3 days. To ensure adequate regeneration and sufficient construct expression, electrophysiological or behavioral experiments were performed after 10 to 14 days.

#### Brain slice recordings

Acute sagittal 250 μm thick cerebellar brain slices of Bl/6 mice were prepared 14 days after virus injection using a vibratome (VT1000S, Leica) in complete darkness. Mice were first deeply anesthetized with isoflurane and decapitated. The cerebellum was removed and cut in ice-cold cutting solution containing 87 mM NaCl, 2.5 mM KCl, 0.5 mM CaCl_2_, 7 mM MgCl_2_, 1.25 mM NaH_2_PO_4_, 25 mM NaHCO_3_, 10 mM D(+)-Glucose and 75 mM Sucrose bubbled with 95% O_2_ and 5% CO_2_. Brain slices were then incubated for 60 min at 37°C in external solution (125 mM NaCl, 2.5 KCl mM, 2 mM CaCl_2_, 1 mM MgSO_4_, 1.25 mM NaH_2_PO_4_, 26 mM NaHCO_3_, and 20 mM D(+)-Glucose with continuous oxygenation 95% O_2_ and 5% CO_2_. Patch electrodes with a resistance of 3–5 mΩ were filled with internal solution (125 mM Potassium Gluconate, 10 mM HEPES, 4 mM NaCl, 2 mM MgCl_2_, 0.2 mM EGTA, 4 mM Mg-ATP, 0.4 mM Na-GTP, 10 mM Tris-Phosphocreatine and 5 mM lidocaine N-ethyl bromide (QX-314; Tocris Bioscience; pH 7.3, 280mOsm). mCherry-positive Purkinje cells were visually identified under a 40× objective attached to an upright microscope (BX51WI, Olympus) and light pulses (1.6 mW mm^−1^ light intensity) of 560 nm wavelength generated by a Polychrome V (TILL Photonics) were used. Whole-cell patch-clamp recordings were measured using an EPSC10 amplifier (HEKA), digitized at 10 kHz and low pass-filtered at 3 kHz. Purkinje cells were clamped with a holding potential of −60 mV and by using a second glass pipette, positioned in the molecular layer of the cerebellum, PF were stimulated. The recording started with a 5 min baseline protocol, followed by 5 min LTD induction and ended with a 20 min post-LTD measurement. For the baseline and post-LTD measurements, a paired pulse stimulation of PF (ISI = 100 ms) was used every 30 s. No significant change in the paired-pulse ratio before and after LTD induction were found. Cells that showed a change in the leak current > 1 nA during the long recordings were excluded from the analysis. To visually induce LTD, PCs were illuminated with 470 nm light pulses (1.8 mW/mm^2^) with a duration of 400 ms and was combined with 75 single pulse PF stimulation at 0.5 Hz. For blocking experiments, Linsitinib (OSI-906; 75 nM), U73122 (5 μM), or PD98059 (50 μM) was additionally added to the external solution. To pharmacologically induce LTD PCs were voltage-clamped at −60 mV and PFs stimulated. After 5 min baseline measurement, insulin (500 nM) was added into the external solution and post-recorded for another 20 min. For the analysis of LTD of EPSCs, the mean responses of the cells before and after stimulation were compared: 0–20 min for light and depolarization, and 10–30 min for insulin stimulation. For the experiments involving additional blockers response from 0 to 10 min (light and depolarization) or from 10 to 20 min (insulin) after stimulation were averaged. PatchMaster software (HEKA) was used for data acquisition and MATLAB for offline analysis.

#### Western Blots

To prepare Western Blots acute sagittal 250 μm thick cerebellar brain slices of Bl/6 mice were prepared in red light 14 days after InLOV or mCherry virus injection as described above. The slices were either illuminated with blue light, incubated with insulin (500 nM) as a positive control or directly frozen with liquid nitrogen without light contact. Tissue slices were homogenized in 100–200 μL of RIPA buffer (Cell Signaling) supplemented with+ PhosphoSTOP and ProteaseSTOP (Thermo Fisher) in Precellys Tissue Homogenizer (Bertin) cooled to 0-4C. Tissue homogenates were then incubated on ice for additional 20 min, centrifuged in pre-cooled table centrifuge for 15 min/13000 rcf and supernatants containing the protein were collected. 20 μg total protein lysate (in RIPA) were used and western blot performed according to standard laboratory procedures (semi-dry blotting). Following primary antibodies were used: (all in 2% BSA/TBS-T (Tween 0.1%); β-Actin (Merck, Sigma A1978, 1:1.000, mouse), intracellular insulin receptor domain (IRb), (Cell Signaling #3025S, 1:1.000, rabbit), pIR b (Cell Signaling #3024S, 1:500, rabbit), pAkt (Cell Signaling #9271, 1:1.000, rabbit), pErk1/2 (Cell Signaling #4376S, 1:1.000, rabbit). Band intensities were quantified by densitometry using ImageJ: integrated density of pIRβ expression was measured and normalized to loading control (β-Actin) in the same lane.

#### Kinome array profiling of Ser/Thr kinase activity

Brain slices were prepared and treated and frozen directly in liquid nitrogen as described for Western blots. Sample were lysed in a lysis buffer containing phosphatase and protease inhibitors (1:100), incubated on ice for 30 min, centrifuged, and the resulting supernatant divided between three vials and stored at −80°C. The kinome activity profiling platform, PamStation12, was used to measure serine/threonine kinome activity using the STK PamChip (PamGene International). 2 μg of total protein lysate is applied per array. All replicates (control no treated, insulin or light treated brain slices) were measured in triplicate balanced over 3 PamChips (2 runs) with the STK system. Data was analyzed using the BioNavigtor software (powered by Tercen) with STK workflow analysis protocol. Phosphosite QC selection for the STK assay is performed by carrying out a nominal CV estimation. Phosphosites with a CV lower than 50% over all measured arrays are included in the downstream analysis. Values after wash (Cycle 124; for the QC phosphosites) are log2 transformed and values below 0 are removed.

To identify significant differences between the conditions at the phosphosite level, the Linear Models for Micro Arrays and Volcano plot were used. The Upstream Kinase Analysis (UKA) algorithm was used to predict differential kinase activity in the insulin or InLOV condition compared to control. Visualization of the data on a phylogenetic tree was done with the wab-based tool CORAL kinome Tree (http://phanstiel-lab.med.unc.edu/CORAL).

#### Histology and antibody staining

To fixate the brain tissue for immunohistochemistry, the transcardial perfusion method with PBS and 4% paraformaldehyde (PFA; Sigma Aldrich, Taufkirchen, Germany) in PBS (pH 7.4) was used. The fixed and removed brains were post-fixed for 2 h in 4% PFA and transferred into 30% sucrose (wt/v; Sigma Aldrich, Taufkirchen, Germany) in PBS for dehydration for 1–2 days at 4°C. 35 μm sagittal cerebellar slices were collected (CM3050 S, Leica) and triple washed in 1 x PBS, permeabilized and blocked for 1 h in 0.3% PBS-Triton X-100 (PBST) with 5% normal donkey serum (NDS) at room temperature. Primary Calbindin antibody (1:500) was added in 3% NDS in 0.3% PBST and incubated overnight at 4°C. After washing three times with PBS for 10 min each time, the secondary antibody (donkey anti-mouse conjugated with DyLight 650, 1:500) was applied and incubated for 3 h at room temperature. For the extracellular alpha subunit of the Insulin receptor staining primary Insulin receptor alpha antibody (abcam, #283689) (1:500) was added in 3% NDS in 0.3% TBST and incubated overnight at 4°C. After washing three times with TBS for 10 min each time, the secondary antibody (donkey anti-rabbit conjugated with DyLight 650, 1:1000) was applied and incubated for 3 h at room temperature. Sections were washed again three times with PBS/TBS and embedded in Rotimount Fluocare (CarlRoth, Karlsruhe, Germany).

Images were taken using an inverted Leica TCS SP5 confocal laser-scanning microscope (Leica DMI6000 B, Wetzlar, Germany) or 2P microscope (Ultima 2Pplus, Bruker, USA) The images were processed with ImageJ Fiji.

#### Behavioral experiments

##### Spatial navigation paradigms

All 3–5-month-old mice were handled daily 5 days before testing to minimize stress during experimental procedures. The behavioral experiments were performed in a circular pool (diameter: 96 cm) filled to a depth of 35 cm of water (25 ± 1°C) mixed with white milk powder to hide the platform (diameter: 10 cm) submerged 1.5 cm below water surface. In addition, the walls of the setup were equipped with visual cues (square, lightning, circle, triangle) that are not moved during experiment as an animal’s reference point. The swimming distances of the animals to be tested were recorded using an overhead camera and information such as swimming speed or escape latency was analyzed via EthoVision XT11.5 (Noldus, Information Technology). Immediately before the start of experiments, the implanted mice were coupled to a glass fiber cable for a 10-min blue light optogenetic stimulation (PlexBright LED system, Campden Instruments, UK) while they were free to move around the home cage. The stimulation protocol was adapted from the stimulation protocol used for LTD induction, consisting of 75 pulses with a pulse duration of 400 ms, given at 0.5 Hz frequency. For *Morris Water Maze* paradigm accordingly to Morris 1981,^49^ mice were repeatedly placed into the tank filled with turbid water and had to learn to locate the platform hidden under the water surface to escape. For *Star Maze* paradigm, five alleys that form a central pentagonal ring and five alleys from the vertices of the pentagonal ring were additionally positioned into the tank. In both paradigms, mice were tested on 4 consecutive days and starting locations were randomly assigned based on comparable swimming distances.^50^ The mice had at most 90 s to find the hidden platform which was at the same place every day. 4 trials per mouse were performed per day with an intertrial interval of 90 s. If a mouse did not find the platform after 4 trials, it was placed on it once. For *Path Integration task*, a visible orientation cue was positioned on the platform. The protocol was adapted from Rochefort et al.^40^ On the first day, mice had to learn the way to the platform in bright room lighting (600 lux). 5 trials (60 s with 30 s intertrial interval) per session and a total of 3 sessions per day were performed each. On the second day, sessions 1 and 2 were also performed in bright light, while session 3 was performed in dim light (∼50 lux). On day 3, session 1 and the first trial in session 2 were conducted in dim light, while all the remaining runs were conducted in complete darkness. On the last day, all sessions were performed in the dark, except the first trial which was exercised in in bright room lighting. The data were averaged over the respective trials per day.

### Quantification and statistical analysis

Statistical significance tests were performed with MATLAB or Origin. For all tests, the significance level was set to *p* < 0.05 and indicated together with the *n* values in the figures and/or figure legends. Data are shown as mean ± standard error. Depending on the normal distribution of the data, to compare two independent groups, the two-tailed Student's *t* test, Mann-Whitney U test, Wilcoxon signed-rank test or Kolmogorov-Smirnov test were performed. To compare data of more groups, two-way analysis of variance (ANOVA) or Kruskal-Wallis tests with subsequent post hoc tests for detailed comparisons were employed. Statistical details of the experiments can be found in the figure legend. In figures, asterisks denote statistical significance marked by ∗ *p* < 0.05, ∗∗ *p* < 0.01, ∗∗∗ *p* < 0.001, and “n.s.” indicates no statistical significance.
